# Occurrence of *Penicillium verrucosum*, ochratoxin A, ochratoxin B and citrinin in on-farm stored winter wheat from the Canadian Great Lakes Region

**DOI:** 10.1371/journal.pone.0181239

**Published:** 2017-07-27

**Authors:** Victor Limay-Rios, J. David Miller, Arthur W. Schaafsma

**Affiliations:** 1 Department of Plant Agriculture, University of Guelph, Ridgetown, Ontario, Canada; 2 Department of Chemistry, Carleton University, Ottawa, Ontario, Canada; Tallinn University of Technology, ESTONIA

## Abstract

The occurrence of *P*. *verrucosum* and ochratoxin A (OTA) were surveyed for 3 and 4 years, respectively. A total of 250 samples was collected from an average of 30 farms during the 2011, 2012, 2013 and 2014 winter seasons. Most storage bins surveyed were typically 11 m high round bins made of corrugated, galvanized steel, with flat-bottoms and conical roofs. Samples of clumped grain contained the most *P*. *verrucosum* (*p*<0.05, n = 10) followed by samples taken from the first load (n = 24, mean = 147±87 CFU/g) and last load (n = 17, mean = 101±77 CFU/g). Five grain samples (2.2%) tested positive for OTA, citrinin and OTB at concentrations of 14.7±7.9, 4.9±1.9 and 1.2±0.7 ng/g, with only three samples exceeding 5 ng/g. Grain samples positive for OTA were related to moisture resulting from either condensation or migrating moist warm air in the bin or areas where precipitation including snow entered the bin. Bins containing grain and clumps contaminated with OTA were studied in detail. A number of statistically-significant risk factors for OTA contamination were identified. These included 1) grain clumps accumulated around or directly under manhole openings, 2) debris and residue of old grain or grain clumps collected from the bin walls or left on storage floor and augers and 3) grain clumps accumulated around side doors. Even when grain enters storage below the 14.5% threshold of moisture, condensation and moisture migration occurs in hotspots in modern corrugated steel storage bins. Hot spots of OTA contamination were most often in areas affected by moisture migration due to inadequate aeration and exposure to moisture from precipitation or condensation. Further, we found that the nature of the condensation affects the nature and distribution of small and isolated areas with high incidence of toxin contamination and/or *P*. *verrucosum* prevalence in the grain bins examined.

## Introduction

Winter wheat has become an important crop for Ontario comprising approximately 73% of Canadian production concentrated in the Southwestern region of the province [1]. In recent years, an increase in on-farm storage capacity has been seen across wheat-growing areas in the USA and Canada, due to rising agricultural commodity prices, and increasing farm sizes, facilitating infrastructure investments [2]. In Ontario alone, on-farm wheat stocks in December 2011 comprised 41% of the total wheat harvest declining to 14% in March and only 5% in July [3]. Maintaining grain quality and preventing mycotoxin formation in storage are crucial for individual farmers and the grain industry in general.

In northern temperate growing areas, ochratoxin A (OTA) is produced by *Penicillium verrucosum* Dierckx during grain storage [4]. OTA often co-occurs with ochratoxin B (OTB) and citrinin in cereal grains [5]. OTA has been classified as a possible human carcinogen [6]. Discovered in a laboratory culture of *Aspergillus ochraceous* Wilhelm in 1965, OTA was reported as a natural contaminant in stored grain destined for animal feed in a maize sample collected in Iowa, USA [7, 8]. It was detected three years later in a moldy red spring wheat sample collected in Alberta, Canada [9]. In Ontario, it was first reported in white beans in 1972 and subsequently in suspect maize and mixed grain samples [10]. Produced by several species of fungi, ochratoxin is found in several commodities (cereals, raisins, coffee). OTA has been shown to be quite common in cereals. For example, 37% of 1,907 samples randomly selected from Canadian wheat domestic and export shipments between 2010 to 2012 contained quantifiable levels of OTA all below the EU regulation [11]. Small concentrations of OTA were reported in ~40% of wheat-based breakfast cereal and most pasta samples at the retail level in Canada [12, 13]. In the USA, 52% of breakfast cereal and snacks collected from 2012 and 2013 tested positive for OTA [14].

The major contributors of OTA exposure in the Canadian population, based on consumption rates, are wheat-based foods [15]. In order to reduce its exposure, various countries including the European Union have set regulations for OTA content in beverages, food and animal feed [16]. Health Canada has proposed maximum limits for the presence of OTA in various food commodities including a 5 ng/g maximum level for raw cereal grains and 0.5 ng/g maximum limits for baby foods and processed cereal-based foods for infants and young children [17]. The analysis of cereal-based foods is particularly challenging as the aforementioned limit approaches our current level of detection (0.3 ng/g) obtained using an LC-MS/MS system. The Canadian Food Inspection Agency has already applied the proposed limits through a market basket survey of cereal-based baby foods resulting in a voluntary product recall in Ontario in late 2009 [18]. Considering a 2-kg wheat sample from a grain lot, almost all (95%) of the variability in the analysis of OTA at the ng/g level was due to sampling; the remainder was due to the analytical and sample preparation steps [19]. Aside from the costs, the extreme variation of OTA in grain makes analysis of grain leaving farms impractical. This has resulted in the value chain erring on the side of caution, greatly increasing the likelihood of economic harm to grain producers. False positives were identified as a major problem of rapid immunoassay kits commercially available in Europe for OTA determination in cereals [20]. Samples that were identified as positive needed to be confirmed using an expensive reference method. This disadvantage coupled with the heterogeneous nature of OTA in grains has created angst in the cereal value chain resulting in increased surveillance costs with dubious certainty. Until more reliable and cost-effective sampling and analytical methods are available, OTA must be managed in winter wheat through the reduction of risk primarily at the origin of the value chain, on farm and in storage. Regional [21] and temporal [12] variations in OTA levels in human blood and cereal products has been observed, respectively, indicating the importance of multiyear surveillance but data for Canadian grains are scarce.

Managing the potential contamination of grain with OTA requires an understanding of the occurrence of OTA-producing fungi as well as the conditions post-harvest and during storage that are conducive to toxin accumulation in grains. The objectives of the current study were: 1) to conduct an annual survey of post-harvest and on-farm storage conditions related to the occurrence of *P*. *verrucosum* and related mycotoxins in winter wheat grain collected from on-farm storage; 2) to study the relationship between mycotoxin contamination and *P*. *verrucosum* occurrence in winter wheat grain; 3) to identify potential critical on-farm storage control points for *P*. *verrucosum* inoculum incidence and mycotoxin accumulation; 4) to identify conditions conducive to OTA accumulation in winter wheat grain on farm and suggest best management practices from this information.

## Materials and methods

### Sample collection and preparation

#### On-farm storage bin survey

**Fig 1:**
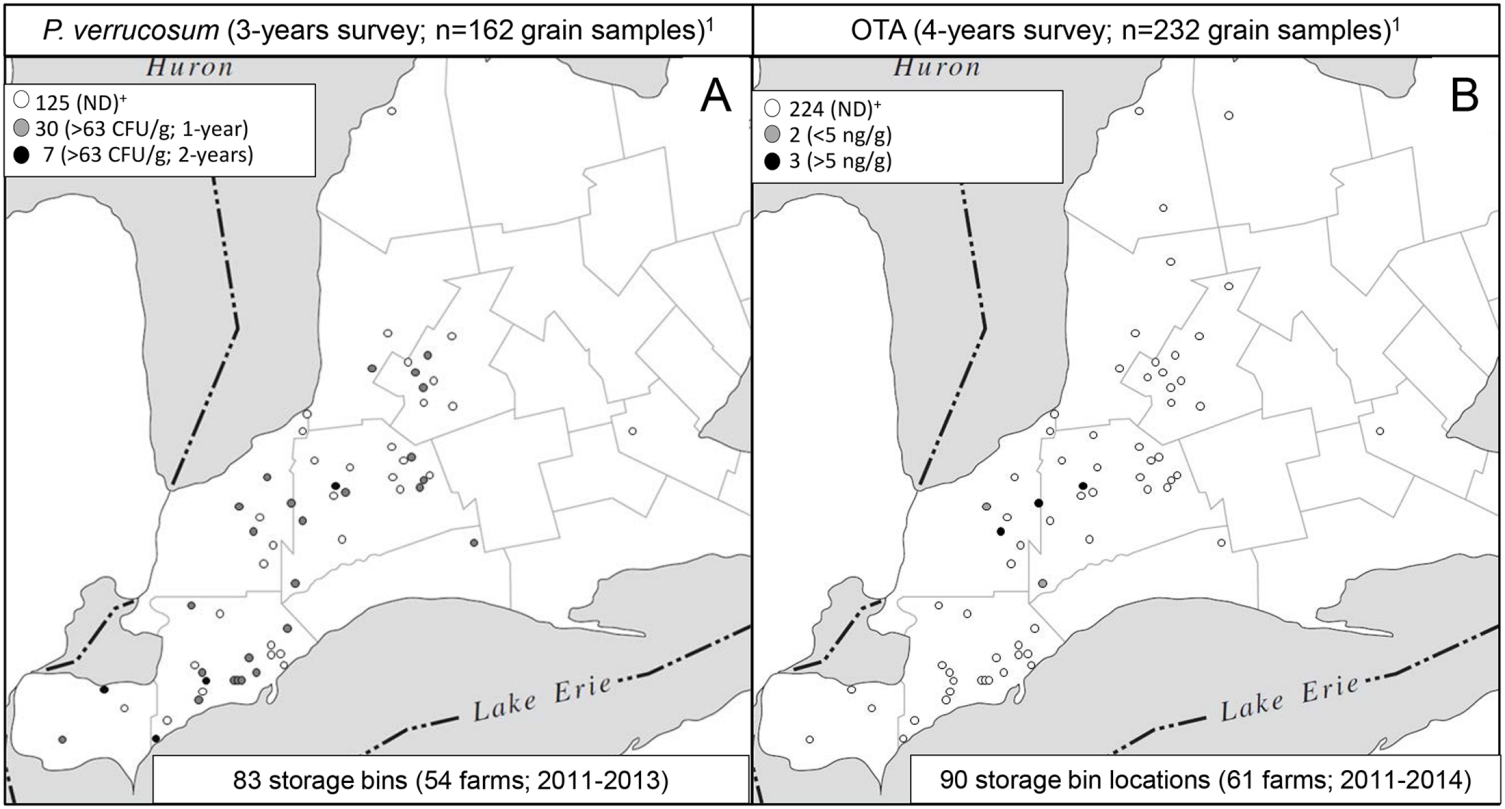
Geographic distribution of *P*. *verrucosum* (A) and OTA (B) occurrence in winter wheat grain collected from on-farm storage bins across Southwestern Ontario, Canada. ^1^Total grain samples collected. Open circles and dots represent individual on-farm storage bin operations. ND: bin operations where grain tested negative for *P*. *verrucosum* and/or OTA. CFU determined by dilution plating on DCYES media with a LOD: 63 CFU/g; OTA, LOD: 0.3ng/g determined by LC-ESI(+)-MS/MS.

A 3- and 4-year annual survey of the occurrence of *P*. *verrucosum* and OTA, respectively, targeting on-farm winter wheat storage bins was conducted at various locations across Southwestern Ontario from 2011 to 2014 (Fig 1).

Farmers that harvest, dry and store their own grain were selected for this study by volunteerism in response to an appeal through grain grower organizations (Grain Farmers of Ontario) and government extension (Ontario Ministry of Agriculture Food and Rural Affairs). In this study humans were not the subject. Instead, we surveyed agronomic practices for which a formal ethical review was not required. No personal information on willing participants was or will be disclosed including GPS coordinates.

Information sheets were completed by each farmer and used to document agronomic, post-harvest and storage practices. A total of 40, 57, 65 and 70 grain samples were collected from 31, 34, 26 and 29 farms (37, 36, 37 and 40 storage bins) during the 2011, 2012, 2013 and 2014 winter seasons. All samples were collected by field technicians in coordination with each farmer using standardized protocols (following). In order to establish the potential source of mycotoxin contamination and to standardize samples across bins, grain samples were classified based on collection source and timing (before, during and after storage and bin out-loading onto trucks) as described in Table 1.

**Table 1:** Classification of grain samples based on collection source and timing (before, during and after storage and bin out-loading).

Source	Type	Description	Timing			P. v			OTA		
			(grain out-loading)			2011–13^2^			2011–14^3^		
			before	during	after	N^4^	n^5^	(%)^5^	N^4^	n^5^	(%)^5^
UntoSuched surface	Static	From the centre top of bins left untouched during storage	X			83	22	(26.5)	108	3	(2.8)
Disturbed surface	Static	From the centre top of bins where grain had been partially removed during storage	X			18	3	(16.7)	29	0	(0.0)
First loads	Dynamic	From the first grain augered out of the bin		X		24	6	(25.0)	40	1	(2.5)
Intermediate loads	Dynamic	Aggregated grain samples from the auger collected during out-loading		X		20	2	(10.0)	24	0	(0.0)
Last loads	Static	From the top of grain piles from the last loaded wagon			X	17	4	(23.5)	31	1	(3.2)
Total grain samples^1^						162	37	(22.2)	232	5	(2.2)
Grain crumps	Static	Moldy grain clumps found inside the bins			X	10	7	(70.0)	18	10	(55.6)

^1^Total grain samples collected;

^2^samples collected from 2011–2013 were tested for *P*. *verrucosum* using dilution plating on DCYES media with a LOD: 63 CFU/g;

^3^samples collected from 2011–2014 were analyzed for OTA using a LC-ESI(+)-MS/MS system, LOD: 0.3ng/g;

^4^N: total number of sample collected;

^5^n: positive samples and (%) of positive samples.

The initial aim of this study was to monitor the undisturbed surface of the grain mass before any grain was removed for shipping (bin out-loading). This was the main source of samples. For sampling, a cleaned commercial grain probe (extendable to 6 m length, 3 cm diameter, aluminum) was inserted to approximately 60-cm depth into the top of the bulk grain surface at the center of the bin. Around 12 kg of grain was aspirated out through the probe, using a modified 6.0 HP drum vacuum cleaner with a storage capacity of 45.5L (Shop Vac Corp., Chicago, IL) and transferred to a new, unused cotton sack (38 cm wide by 71 cm long). The vacuum was cleaned with methanol, and a methanol-soaked disposable cloth was passed through the length of the probe several times between each sampling location. In some cases, we arrived at storage bins where the grain surface was disturbed resulting from unloading or inter-bin grain transfer during storage. These samples were also collected but considered separately. Another source of samples was those collected from the first grain leaving bins during the first truck load taken from the bin (first loads samples) generally taken when the center top of the bin was inaccessible. These samples were collected by using a dip cup (630 mL) fixed on a pole inserted into the moving stream of grain at 1 min intervals for 15 min until approximately 12 kg of grain was obtained. The cup was cleaned with methanol between each sampling location.

A further source of samples was those obtained from farm stores when the first load and perhaps several loads had already been shipped, before we arrived (intermediate load samples). These samples were taken as described above. Finally, in some cases we were only able to sample from the last load leaving the bin. In this situation samples were collected from the top of the last loaded grain wagon or truck (last load samples) in the same way grain was collected from the surface of the bin. During the last three years of the survey, whenever grain clumping was observed in the bin after shipping, grain was also collected directly from clumps, and the clump location and conditions were documented. Additionally, each time a grain or grain clump sample tested positive for OTA, the storage bin was studied in detail with emphasis on measuring OTA, OTB and citrinin relative occurrence at different location in the bin. A total of 6 storage bins were sampled for grain clumps found near manhole opening and door frames, debris and old grain found in augers, on floor and on walls and grain collected from different areas in the bin.

In the first year of the survey, individual grain samples were split into 10 and 2 kg fractions using a continuous splitter, placed into two new unused cotton sack and shipped to the Canadian Grain Commission’s Grain Research Laboratory and University of Guelph Laboratory Services, respectively, for independent determination of OTA contamination by LC-MS/MS. Ground and homogenized samples were sent back to our laboratory in Ridgetown for further mycotoxin analysis and mycological determination. Samples from the second and third years were analyzed in Ridgetown. All samples were transported on the same day of collection, evaluated for visual insect damage, mold presence and moisture content measured with a Motomco Automatic Grain Moisture Meter (Model 919), and then stored in an air ventilated cold room at 2°C and 40–50% RH until analysis. The 12-kg grain samples were blended for 5 min using a clean electric stainless steel cement mixer. Twenty 100-g portions were removed from the rotating mixer to form a 2-kg composite sub-sample. The entire sub-sample was then ground into 3 portions using a Romer sub sampling mill (Model 2A, Romer Labs., Union, MO). One portion was ground further to a particle size of less than 850 microns using a M2 Stein mill (Fred Stein Lab, Inc., Atchinson, KS). A 100-g sub-sample was conserved in a 500-mL wide-mouthed polypropylene bottle sealed with screw cap for mycotoxin analysis. Two 0.5-g sub-samples were conserved in separate 50- mL centrifuge tubes for *P*. *verrucosum* determination.

### Mycological analysis

#### *P*. *verrucosum* isolation, quantification and identification

Preliminary studies were made to select an optimal medium for isolation of *P*. *verrucosum* from grain samples. The final choice was Dichoran chlorotetracycline yeast sucrose glycerol media (DCYES: 20 g of yeast extract, 150 g of sucrose, 20 g of agar, 0.5 g of MgSO4, 0.05 g of chloramphenicol, 0.002 g of dichloran, 0.05 g of chlorotetracycline, 0.01 g of ZnSO4, and 0.005 g of CuSO4 per liter) [22]. DCYES plates had sufficient growth (>2 mm in diameter) in 6 d which was faster compared to the other recommended media (10–14 d) with a water activity (aw) of 0.9930 ± 0.003 (measured in triplicate in 10-day-old cultures using an AquaLab 4TE-DUO, Decagon Devices Inc., USA). Plates were incubated in the dark at 25° for 7 d. Three strains of *P*. *verrusosum* (no, medium-, and high- OTA producers with strain numbers C27-1, C924-2 and C258-1, respectively) were used for method validation and were obtained from the Canadian Grain Commission, Winnipeg, Manitoba.

*P*. *verrucosum* colony-forming units (CFU/g sample) were estimated from ground grain and debris samples by serial dilution plating. This approach was tested using a winter wheat sample collected from the first bin that had grain tested positive for OTA in our 2011 on-farm storage survey. After plating (see below), presumptive isolates of *P*. *verrucosum* were counted based on colony morphology (terracotta to reddish-brown on reverse). Randomly selected colonies were tested for the production of OTA and citrinin by thin layer chromatography (TLC) based on TEF (toluene/ethyl acetate/90% formic acid, 5/4/1, v/v/v) and CAP (chloroform/acetone/2-propanol, 85/15/20, v/v/v) eluting solutions using a silica gel C18 pre-coated aluminum backed plates with fluorescent indicator UV254 (Macherey-Nagel, Duren, Germany), and observed under a 365 nm UV light [23]. All 3 *P*. *verrucosum* standard strains produced citrinin and two produced OTA. For further verification, LC/MS-MS analysis for OTA, citrinin and OTB was conducted on colonies positive for OTA and/or citrinin as determined by TLC. The first 7 cultures evaluated produced OTA, citinin and OTB were sent to the Agriculture and Agri-Food Canada Laboratory in Ottawa for identification of *P*. *verrucosum*. This was done using sequence-specific-primer-PCR methods targeting the β-tubulin gene using Bt2a and Bt2b primers [24] as described by Samson *et al*, 2004 [25]. They reported that all cultures were authentic *P*. *verrucosum* and given the following ID: numbers KAS 4254, KAS 4255, KAS 4256, KAS 4257, KAS 4259, KAS 4260 and KAS 4262. The genome of KAS 4260 has been fully sequenced and the colony has been deposited by Dr. Keith Seifert in the Canadian Collection of Fungal Cultures (accession number DAOM 242724) located at the Eastern Cereal and Oilseed Research Centre, Agriculture and Agri-Food Canada, Ottawa, Ontario.

The final protocol modified in our study was as follows: two 0.5-g ground subsamples, from each sample described above were diluted 1:100 (w/v) in double-distilled water by shaking for 30 min in separate 50-mL sterile polypropylene centrifuge tubes. A 200-μL suspension was spread uniformly on the surface of DCYES media contained in a sterile 100-mm disposable petri dish, using a sterile glass stir rod, and the inoculated dishes were kept for approximately 7 d in darkness at room temperature. Each sub-sample was replicated four times resulting in 8 replications per sample. The number of presumptive *P*. *verrucosum* colonies appearing on plates was counted yielding a detection limit of 63 CFU/g. In some cases, if plates appeared over grown with colonies of other fungal species, hindering the growth of *P*. *verrucosum*, the sample was re-plated at increasing dilution factors to a maximum of 1:1000000 (w/v) or 625000 CFU/g. For mycotoxin profiling, up to 5 colonies per sample were selected randomly and transferred to DCYES media and grown for another 10 d for qualitative and quantitative mycotoxin profiling using TLC and/or LC-MS/MS. Quantitative levels of OTA, OTB and citrinin were produced by all 5 presumptive isolates randomly isolated from each sample. Thereafter presumptive colonies were considered as *P*. *verrucosum*.

### Mycotoxin analysis

#### Chemicals and reagents

Methanol, acetonitrile and water of LC-MS grade were obtained from J.T. Baker (Phillipsburg, NJ, USA). Ammonium formate and formic acid of LC-MS grade, and glacial acetic acid and ammonium acetate of HPLC grade, were purchased from Fisher Scientific (Oakville, ON, Canada). OTA, OTB and citrinin standard solutions and isotope-labelled internal standard U-[^13^C_20_]-Ochratoxin A (U-[^13^C_20_]-OTA) were purchased from Biopure (Tlln, Austria) and dissolved in acetonitrile. All other reagents were of analytical grade and obtained from Fisher Scientific (Oakville, ON, Canada).

#### Extraction from grain

A 100-g sub-sample was placed in a 500-mL wide-mouthed polypropylene bottle with screw cap and extracted with 400 mL of acetonitrile/water/acetic acid (79/20/1, v/v/v) by shaking for 90 min using a MAX Q 2000 Thermo Scientific rotary shaker (Sulyok et al., 2006). The extract was transferred to a polypropylene centrifuge tube, spun for 2 min at 3000 rpm in a centrifuge with a radius of 15 cm. A 75-μL aliquot of supernatant was transferred to a 10-mL glass vial, dried down using a Pierce Reacti-VapTM III machine at 40°C, reconstituted in 150 μL of acetonitrile/water (50/50, v/v) solution and then transferred to a 400-μL glass vial insert in a 2-mL amber glass vial. The reconstituted sample extract was spiked with U-[^13^C_20_]-OTA internal standard to a concentration of 1 ng/mL. A 50-μL aliquot was then injected into the HPLC system, with an eluent flow rate of 1 mL/min. Eluent from the HPLC was then split by a 50:50 flow splitter, and 25 μL was introduced to the ESI-MS/MS.

#### Extraction from mycelium

Fungal mycelium mass was added to acetonitrile/water/acetic acid (79/20/1, v/v/v) extraction solvent in a ratio of 1:4 (w/v), respectively, and then homogenized with a Fisher Scientific Pulsing Vortex Mixer for 5 min. A 100-μL sample was added to 900-μL methanol/water/acetic acid (10/79/1, v/v/v) solution, centrifuged for 2 min at 3000 rpm. A 500-μL aliquot of supernatant was transferred into a test tube, evaporated, reconstituted with 1000 μL of an acetonitrile/water (50/50, v/v) solution, then transferred to a 400-μL glass vial insert in a 2-mL amber glass vial, and a 25-μL aliquot was introduced to the LC-MS/MS. The reconstituted sample extract was spiked with U-[^13^C_20_]-OTA internal standard to a concentration of 1 ng/mL.

#### Mycotoxin determination

OTA, OTB, and citrinin detection and quantification were performed with an Ionics EP 10+ modified API 365 triple quadrupole mass spectrometer (LC-MS/MS; AB SCIEX, Concord, ON) system equipped with an electrospray ionization (ESI) source. The Agilent 1100 Series HPLC system consisted of a G1312A binary pump, G1316A thermostat column oven, G1322A degasser (Agilent Technologies, Santa Clara, CA), and a HTC Pal autosampler (CTC Analytics, Zwingen, Switzerland) equipped with a 100-μL injection syringe. A 150-mm Gemini C18 reverse phase column with 4.6-mm internal diameter (i.d.), and particle size of 5 μm fitted with a C18 4x3-mm i.d. security guard cartridge (Phenomenex, Torrance, CA) were used to complete separation [26]. Eluent A contained 5 mM of ammonium formate, and 0.1% formic acid in water. Eluent B contained 100% acetonitrile. Separations were conducted at a flow-rate of 1mL/min using 100% eluent A for the first 2 min followed by a linear gradient from 0 to 100% of eluent B over the next 12 min, then a hold time of 3 min at 100% B and finally linearly changed back to 100% eluent A over 10 sec and re-equilibrated at 100% A for an additional 50 sec. The total run time for each sample was 18 min. ESI-MS/MS was performed in multiple-reaction-monitoring (MRM) mode in positive polarity. The instrument settings were as follows: source temperature 550°C, nitrogen curtain gas 80 psi, nebulizer gas setting 8, collision gas setting 2, and ionization voltage 5000V. The nitrogen gas of 99% purity was supplied by a Source 5000 Tri Gas Generator (Parker Balston, Haverhill, MA).

#### Analytical performance

The parameters used in the detection and quantitation of OTA, OTB and citrinin were obtained via direct infusion of (6 ng/μL) solutions at 10 μL/min into the ESI-MS/MS. Potentials producing the most intense response to their respective toxins were selected for the method (Table 2).

**Table 2:** HPLC-ESI-MS/MS optimized instrument parameters and recovery (%), repeatability (SD%), LOD and LOQ of OTA, OTB and citrinin spiked in winter wheat flour.

Parameter	Unit	OTA	OTB	citrinin	OTA ^13^C_20_ ^1^
^t^R^2^	(min)	12	11.1	11.6	12
Precursor ion^3^	(m/z)	404.2 [M+H]^+^	370.1 [M+H]^+^	251.1 [M+H]^+^	424.2 [M+H]^+^
DP^3^	(V)	44	37	22	44
Product ion^3^	(m/z)	**239.0**/358.0	**205.1**/103.0	191.0/205.2/**233.1**	250.0/377.3
CE^3^	(V)	34/20	32/76	33/33/33	31/19
CXP^3^	(V)	17/36	17/17	17/17/17	33/34
Recovery ±SD^4^	%	104±5	88±7	105±4	
Linearity range^5^	ng/g	0.1–4.0	0.1–4.0	0.1–4.0	
LOD±SD^5^	ng/g	0.3±0.1	0.5±0.2	0.6±0.1	
LOQ±SD^5^	ng/g	0.8±0.3	1.1±0.4	1.4±0.3	

^1^Internal standard;

^2^ Retention times obtained from 4 ng/mL solutions injected into the HPLC;

^3^Determined by direction infusion of 6 ng/mL toxin solutions in a 50/50 Eluent A/B mixture; Quantifier ion data in bold;

^4^Recovery was determined by spiking mycotoxins at 0.5ng/g and equilibrated for 3 days at 40°C before extraction (n = 3);

^5^Matrix-matched calibration curve (n = 6) was employed to determine level of detection (LOD) and quantitation (LOQ).

The method was validated for wheat and maize matrices. Concentrations were determined by using matrix-matched calibration curves at 7 concentration levels from 0.06 to 4.00 ng/mL in addition to a double blank (matrix extract) and a blank (matrix extract with IS) samples. U-[^13^C_20_]-OTA internal standard, (1 ng/mL) was used in this study to compensate for variation of signal intensities due to matrix effect [27]. Instrument control, parameter optimization, data collection and peak integration were performed using Analyst 1.5 software (Applied Biosystems, Foster City, CA). LOD and LOQ were calculated as the mean height of the noise signal obtained for the blank plus 3 and 10 times their standard deviation around the analyte retention time, respectively [28]. LOD and LOQ were 0.3, 0.5, 0.6 ng/g and 0.8, 1.1, 1.4 ng/g for OTA, OTB and citrinin, respectively. Percentage of recovery for each toxin was determined by spiking, in triplicates, 2.5 ng of analytical standards to 5 g of finely ground clean grain samples and allowing them to evaporate and equilibrate with the matrix for 3 days at 40°C in darkness before extraction. Recovery of OTA, OTB and citrinin spiked at 0.5 ng/g was 104%, 88% and 105%, respectively (Table 2).

### Statistical analysis

All data were analyzed using SAS 9.4 software (SAS Institute Inc, Cary, NC). Data were tested for normality with the Shapiro-Wilk test (PROC UNIVARIATE). The CFU data of *P*. *verrucosum* from the 2011–2013 survey of on-farm stored winter wheat grain were transformed to ln(x+1) prior to analysis. The PROC MIXED module with the PARMS statement and the NOITER option was used to implement restricted maximum likelihood (REML) to estimate variance components [29]. Sources of variation included year and farm location nested within years as random effects, and sample source, wheat classes, bin capacity, weeks in storage and grain moisture content at sampling as fixed effects. To reduce small sample bias, the degrees of freedom of the fixed effects F-test were adjusted for statistical dependence using the Kenward—Roger method. All tested interactions that were not statistically significant were not included in the final model. Multiple regressions were performed with the PROC REG procedure of SAS. The influence of potential on-farm storage control points on OTA, OTB and citrinin incidence was studied on 6 farms with grain and/or grain clumps positive to OTA. Mycotoxin data generated were transformed to ln(x+1) prior to analysis. PROC MIXED was used to implement REML estimates of variance components as described above. Sources of variance included farm location as random effect, and control points in storage and grain out-loading as fixed effects. Main effects were compared using a Tukey-adjusted pairwise comparison with *p* <0.05 considered significant. Unless otherwise stated arithmetic means of non-transformed data ± standard error (SE) are reported.

## Results

### On-farm storage bin Survey

#### Grain sources and characteristics

A total of 59 unique farms were surveyed during 2011–2013. Of these, 72% indicated spraying triazole fungicide for controlling *Fusarium* head blight during wheat heading, 13% also applied strobilurin fungicides for foliar diseases early in the season at 4th leaf and/or flag leaf stage of development. Approximately, 67%, 30% and 3% of harvested grain originated from fields following a zero-tillage, minimum tillage and both zero and minimum tillage, respectively. The relationship between pre-harvest and agronomic factors is the subject of a future paper.

Most (77%) of the samples collected from bins were of the soft red winter wheat (SRW) class followed by, soft white winter wheat (SWW, 7%), hard red winter wheat (HRW, 5%), durum (3%), and blends of different classes (8%). The distribution of winter wheat classes in our survey reflects the average area planted with soft red winter wheat, hard red winter wheat and soft white winter wheat in Ontario during the time of this study, which were 83%, 11% and 6%, respectively [30]. Mean grain moisture content at harvest, as reported by farmers, was 15.3%, 14.5% and 13.6% with 51%, 41% and 11% grain samples having a moisture content >14.5% resulting in 16%, 8%, and 11% of farmers using wet grain holding tanks to hold the grain before it was dried for storage in the 2010, 2011 and 2012 cropping season, respectively. In this study, no relationship was found between moisture content measured at harvest by the farmer and *P*. *verrucosum* incidence found in harvested grain (*n* = 131, *p* = 0.7075, *r*^*2*^ = 0.0011) or moisture content measured at sampling after storage (*n* = 120, *p* = 0.9264, *r*^*2*^ = 0.0001).

**Fig 2:**
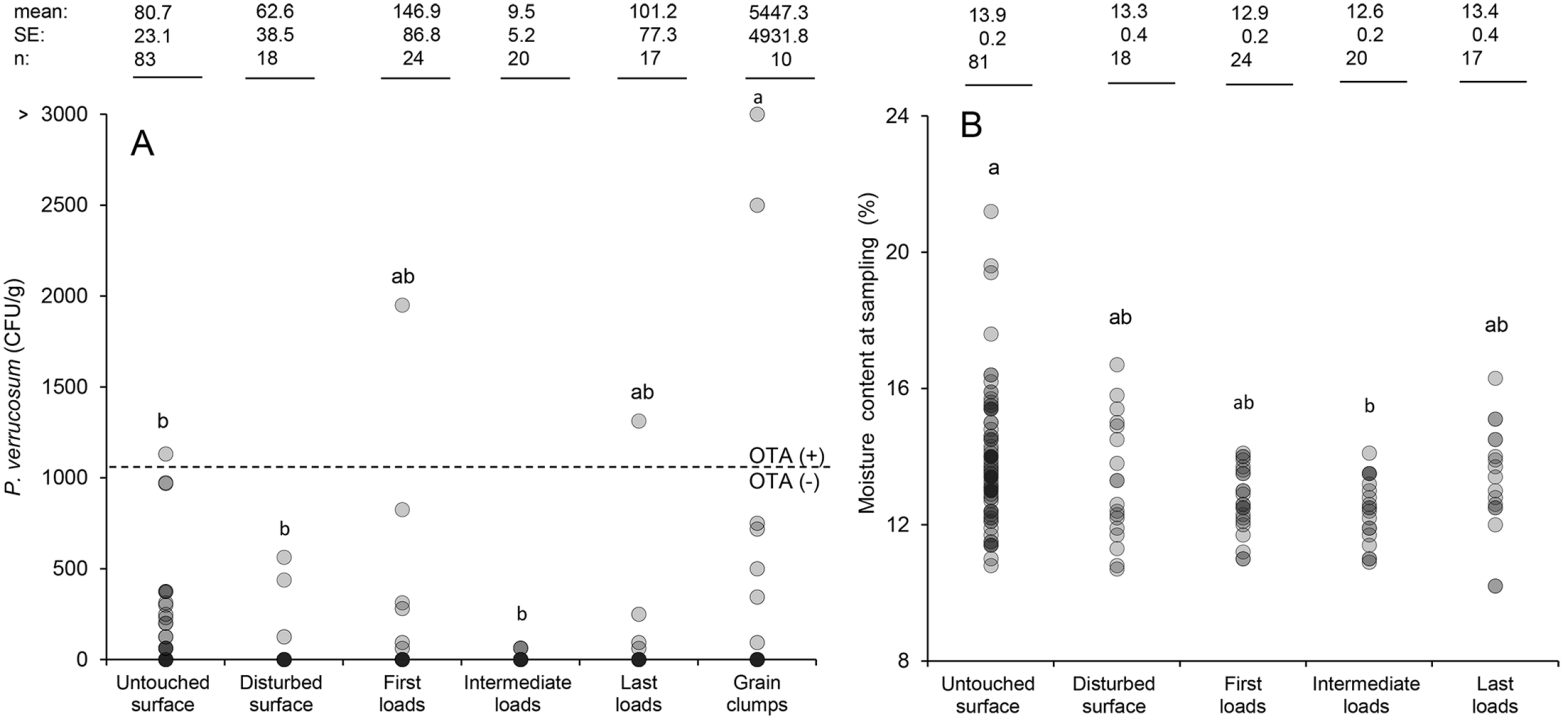
Distribution by sample source of *P*. *verrucosum* (A) and moisture content at sampling (B) found in Ontario winter wheat grain and grain clump stored in on-farm bins and collected from 2011 to 2013. CFU of *P*. *verrucosum* determined by dilution plating on DCYES media; LOD: 63 CFU/g. Means followed by the same letter in the column are not statistically different (Tukey at P<0.05).

Grain moisture content at sampling was significantly higher in samples taken from the surface of untouched bins (*p*<0.05, *n* = 81, mean ± SE = 13.9±0.2%) than aggregated samples taken from the auger during bin out-loading (*n* = 20, mean = 12.6±0.2%). No difference in moisture content was found between those samples collected from the first (*n* = 24, mean = 12.9±0.2%) and last (*n* = 17, mean = 13.4±0.4%) loads and those collected from disturbed surface (*n* = 18, mean = 13.3±0.4%) (Fig 2B).

#### Storage bin characteristics

Of the 83 unique storage bins surveyed during 2011–2013, most (98%) were round bins made of corrugated, galvanized, steel with flat-bottoms and coned roofs with a median capacity of 300 MT (approx. 7 m diameter x 9 m eave height or 11m overall height). All steel bins had aerated flooring equipped with aeration fans, roof ports for top filling, top and bottom man-access doors, bottom unloading ports and ventilation at the eaves. Two of the bins sampled were concrete grain silos. Most (63%) of the bins were smaller with a capacity of less than 400 MT. The remaining 21%, 11%, and 5% of bins had capacities of 1600 MT, 3200 MT and 5000 MT, respectively.

The frequency of aeration for the wheat stored in bins in this study varied from 0, 2, 4 and >6 events in 9%, 17%, 7% and 67% of bins, respectively. Three main aeration periods were reported: 85% were aerated immediately after grain in-loading from harvest; 78% during cool (<15°C) and dry (<60% HR) days in summer and 89% in winter when air temperature outside were below freezing (<-1°C) and dry (<60% HR), respectively.

#### Sample source

Grain samples were classified by two main characteristics: 1) sampling type (either dynamic performed during grain movement at fixed intervals or static when sampling was targeted to specific areas in the bins and wagons) and; 2) sample timing (either sampled before, during and after bin out-loading). Six sample categories were identified including: samples from untouched surface, disturbed pile surface, first load, intermediate loads and last load which made up 47%, 13%, 17%, 10% and 13%, respectively, of a total of 232 grain samples collected during the 2011–2014 survey. Only 18 moldy grain clumps were collected inside the storage bins during the same period. Table 1 summarizes the type and number of samples collected and found positive for *P*. *verrucosum* and OTA during the 2011–2013 and 2011–2014 surveys, respectively.

### *P*. *verrucosum* incidence in grain and grain clumps

The incidence of *P*. *verrucosum* was expressed as number of colony-forming units found per gram of grain sample (CFU/g). Samples containing fewer than 63 CFU/g (lower detection limit) were considered negative. The distribution of the samples testing positive for *P*. *verrucosum* incidence across the geographic area surveyed is shown in Fig 1A.

In the 2011–2013 survey, a total of 40, 57 and 65 grain samples were collected from 31, 34, and 26 farms or 37, 36 and 37 storage bins during the 2011, 2012 and 2013 winter season of which 28%, 25% and 18% were positive for *P*. *verrucosum* at a mean±SD incidence of 383±410, 326±325 and 373±480 CFU/g, respectively. A total of 10 grain clumps was also collected during the 2012 and 2013 winter season of which 7 were positive for *P*. *verrucosum* at a mean±SD concentration of 7782±17163 CFU/g. The highest concentration of *P*. *verrucosum* was found in a 4-kg grain clump sample (49792 CFU/g) collected after grain out-loading near a manhole opening where condensation occurred on the inner side of the metal manhole causing mold growth on the stored wheat, particularly in the grain surface below the opening. All grain (n = 3) and clump (n = 2) samples that tested positive for OTA had an incidence of *P*. *verrucosum* greater than 1000 CFU/g.

There was no difference in the presence of *P*. *verrucosum* between farms (*F* = 0.83, *df*_*n*_ = 52, *df*_*d*_ = 33, *p* = 0.7299) and years (*F* = 0.83, *df*_*n*_ = 2, *df*_*d*_ = 33, *p* = 0.4453) (Table 3). Analysis of variance for a reduced model shows that the presence of *P*. *verrucosum* in grain was affected by where the sample was sourced in the storage bin (*F* = 5.13, *df*_*n*_ = 5, *df*_*d*_ = 74, *p* = 0.0004) followed by moisture content in the grain measured at sampling (*F* = 4.24, *df*_*n*_ = 8, *df*_*d*_ = 47, *p* = 0.0007), weeks in storage (*F* = 3.52, *df*_*n*_ = 14, *df*_*d*_ = 47, *p* = 0.0006) and to a lesser degree storage bin capacity (*F* = 1.71, *df*_*n*_ = 17, *df*_*d*_ = 47, *p* = 0.0751). Wheat classes did not affect *P*. *verrucosum* incidence (*F* = 0.05; *df*_*n*_ = 4, *df*_*d*_ = 74, *p* = 0.9958) (Table 3).

**Table 3:** Analysis of variance for reduced model of the distribution of *P*. *verrucosum* inoculum found in grain and grain clumps (CFU/g) collected in winter 2011–2013 from on-farm storage bins located in Ontario, Canada.

Fixes effect*	*df*_*n*_	*df*_*d*_	*F*	*Pr > F*
Farms	52	33	0.83	0.7299
Years	2	33	0.83	0.4453
Sample source ^1^	5	74	5.13	0.0004
Wheat classes ^2^	4	74	0.05	0.9958
Bin capacity (MT) ^3^	17	47	1.71	0.0751
Moisture at sampling (%) ^4^	8	47	4.24	0.0007
Weeks in storage	14	47	3.52	0.0006

*Data transformed before analysis [ln(X+1)].

^1^Sample source: untouched surface (48%), disturbed surface (10%), first loads (15%), intermediate loads (11%), last loads (10%), grain clumps (6%);

^2^Wheat classes: Soft red winter wheat (77%), hard red winter wheat (5%), soft white winter wheat (7%), Durum (8%), Blend (8%);

^3^Bin capacity: divided in 24 intervals ranging from 100 to 5000 metric tons (MT);

^4^Moisture content was not determined in grain clumps

**Fig 3:**
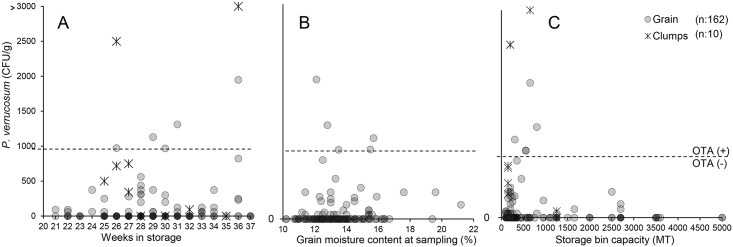
Distribution of *P*. *verrucosum* CFU/g found in grain and grain clumps by weeks in storage (A), moisture content at sampling (B) and on-farm bin size (C) collected in winters 2011–2013. Each gray dot and asterisk represents one grain or grain clump sample, respectively. Bin capacity in metric tons (MT). The dotted line at around 1000 CFU/g separates the OTA positive samples from negative ones. Percentage moisture content (%) was not determined in grain clump samples.

Regression analyses revealed only a very weak relationship between *P*. *verrucosum* incidence (CFU/g) and moisture content measured at sampling (*n* = 160, *p* = 0.0068, *r*^*2*^ = 0.0454), and no relationship with weeks in storage (*n* = 172, *p* = 0.1709, *r*^*2*^ = 0.0110) or storage bin capacity (*n* = 166, *p* = 0.4314, *r*^*2*^ = 0.0038). Two-dimensional scatter plots show that grain and grain clump samples positive for *P*. *verrucosum* tended to be found in grain stored for longer periods of time (Fig 3A), with varying degrees of moisture content at sampling (Fig 3B) and collected from smaller storage bins (Fig 3C).

Significant differences appeared when the data were classified according to where the sample was sourced in the bin (Fig 2). Samples from grain clumps contained more *P*. *verrucosum* (*p*<0.05, *n* = 10, mean±SE = 5447±4932 CFU/g) followed by samples taken from the first load (*n* = 24, mean = 147±87 CFU/g) and last load (*n* = 17, mean = 101±77 CFU/g) augered out of the bins than those samples collected from untouched (*n* = 83, mean = 81±23 CFU/g) and disturbed (*n* = 18, mean = 63±39 CFU/g) grain surfaces and intermediate loads (*n* = 20, mean = 10±5 CFU/g) (Fig 2A).

Grain clumps varied in size from 2–4 kg and were usually found around or directly under manholes, door frames or ice cones on grain surface. These micro pockets of contaminated grain clumps present a significant risk to the value chain particularly for grain milled for infant food. As water leaks or ice thaws it seeps down sides of bin walls and settled in low spots or on floor creating big areas of grain clamping along its path (Table 4). Sampling these clumps was difficult so only debris and old grain found attached to the walls were collected after grain out-loading and included in this analysis (Table 5).

**Table 4:** *P*. *verrucosum* colony counts (CFU/g), mycotoxin occurrence (ng/g) and aeration practices associated with OTA positive grain samples found during the 2011–2014 on-farm storage survey of Ontario winter wheat.

Sampling			Moisture (%) at		Mycotoxins (ng/g)			P. v	Contributing factors*
dd/mm/yy	DIS	Source	Harvest*	Sampling	OTA	OTB	citrinin	(CFU)	
1) 02/20/14	207	Untouched surface^1^	14.5	17.4	44.9	0.6	11.5	4514	No aeration protocol reported. Surface of the grain bulk frozen under a snow cone. Chaff and grain clumps positive for OTA were also found alongside the door, manhole, auger and floor.
2) 02/09/11	209	Untouched surface^1^	15.0	15.7	15.0	4.0	5.0	1014	Aeration when air temperature is cold with low humidity. Water dripping from downspout due to condensation reported.
3) 02/22/12	222	Last loads^3^	16.0	13.4	7.4	0.7	3.3	1313	Non-aerated bin. Sample from top of the wagon (last grain flowing out of the bin) was positive for OTA. The toxin was also found in the residue left on the floor after out loading.
4) 03/25/13	253	First loads^2^	14.0	12.1	4.9	0.7	4.7	2450	Aerated every 6 weeks. Water leak observed from the manhole opening to wall and into the grain. Grain from the top centre of the bin negative for OTA.
5) 02/26/14	216	Untouched surface^1^	13.5	14.4	1.2	0.0	0.1	369	Aerated twice. Grain sample collected from south side under a snow cap. As ice thawed water seeped down sides of bin walls and settled in low spots. Grain clumps positive for OTA found along the wall, manhole, side door and floor.

CFU of *P*. *verrucosum* (P.v.) were determined by dilution plating on DCYES media (LOD: 63 CFU/g). OTA OTB and citrinin were determined by LC-MS/MS with a LOD of 0.3, 0.5 and 0.6 ppb, respectively; DIS: days in storage.

*Aeration practices as reported by farmers. Grain sample collection:

^1^Just before out-loading,

^2^from the first grain streaming out,

^3^on top of the wagon after out-loading was completed.

Untouched surface: grain samples taken from the centre top of undisturbed bins about 0.3 m below the surface.

**Table 5:** Mycotoxin profile of stored winter wheat grain, debris and clumps samples collected from 6 OTA positive bins from 2011–2014 in Southwestern Ontario.

Control point	Sample^1^		OTA (ng/g)						OTB (ng/g)						citrinin (ng/g)					
	type	n	(%)^2^	mean^3^	SE^4^	T^5^	Pr>F^6^	^7^	(%)^2^	mean^3^	SE^4^	t^5^	Pr>F^6^	^7^	(%)^2^	mean^3^	SE^4^	t^5^	Pr>F^6^	^7^
Manhole	Clumps	5	(100)	370.3	198.1	7.2	< .0001	^A^	(80)	47.6	22.1	4.6	0.0001	^A^	(100)	50.6	32.3	7.3	< .0001	^A^
Wall/floor	Debris	6	(100)	80.0	34.0	5.7	< .0001	^AB^	(50)	18.5	12.2	3.0	0.0065	^AB^	(100)	14.9	4.6	7.2	< .0001	^AB^
Auger	Debris	5	(100)	59.7	30.7	5.0	< .0001	^AB^	(100)	11.6	6.1	3.0	0.0062	^AB^	(100)	18.6	8.6	6.2	< .0001	^AB^
Side door	Clumps	4	(100)	46.3	19.0	4.9	< .0001	^AB^	(75)	4.8	3.8	2.1	0.0475	^AB^	(100)	8.9	1.3	5.6	< .0001	^AB^
Untouched Surface	Grain^8^	6	(67)	15.9	8.0	2.9	0.007	^BC^	(33)	0.8	0.6	0.8	0.4556	^AB^	(50)	5.4	2.8	3.4	0.0022	^BC^
First/last loads	Grain	7	(29)	1.8	1.2	0.7	0.514	^C^	(29)	0.2	0.1	0.3	0.7879	^B^	(29)	1.1	0.8	0.7	0.4739	^C^
Disturbed surface/ intermediate loads	Grain	4	(0)	0.0	0.0	0.2	0.818	^C^	(0)	0.0	0.0	0.3	0.7625	^B^	(0)	0.0	0.0	0.8	0.4381	^C^
Variance components	Dfn	Dfd				F	Pr>F					F	Pr>F					F	Pr>F	
Control point	6	24				8.36	< .0001					3.33	0.0157					9.85	< .0001	
Farm	5	24				0.36	0.8949					0.7	0.6495					0.8	0.578	

^1^Samples types: grain clump samples found near manhole opening and door frames, debris and old grain found in augers, on floor and on walls; and grain collected from different areas in the bin.

^2^percentage of positive samples (LOD:0.3 ng/g);

^3^mean and

^4^standard error of untransformed data;

^5^*t* value and

^6^values for the type III error statistics were calculated using ln(X+1) transformation;

^7^Tukey-Kramer multiple comparisons test (P<0.05), rows with the same letter are not significantly different.

^8^one clump was found on the bulk surface.

Because of the low thermal conductivity of grains [31], these clumps were usually restricted to small localized areas without affecting the entire bulk. However, free water caused grain aggregation and even clumping along bin walls forming hang-ups (Table 4). Grain bins tested positive for OTA were studied in details and samples were taken from debris originated from this adherent material (hang-ups) sticking to walls and analyzed for OTA, OTB and citrinin (Table 5).

### OTA incidence

OTA was analyzed in from total of 232 grain samples collected from 90 storage bins belonging to 61 farms during a 4-year survey carried out in Southwestern Ontario. Grain samples were collected from untouched surface of the grain mass (n = 108, 47%), disturbed surfaces (n = 29, 13%), first loads (n = 40, 17%), intermediate loads (n = 24, 10%) and last loads (n = 31, 13%), respectively (Table 1). Of these, only 5 samples tested positive for OTA, citrinin and OTB at a mean±SE level of 14.7±7.9, 4.9±1.9 and 1.2±0.7 ng/g, respectively with a mean incidence of *P*. *verrucosum* of 1,932±728 CFU/g (Table 4). The mean ratio of OTA:OTB was 12:1 ranging from 4:1 to 75:1. In our samples, OTA:citrinin co-occurred at the mean ratio of 3:1 ranging from 1:1 to 12:1.

**Fig 4:**
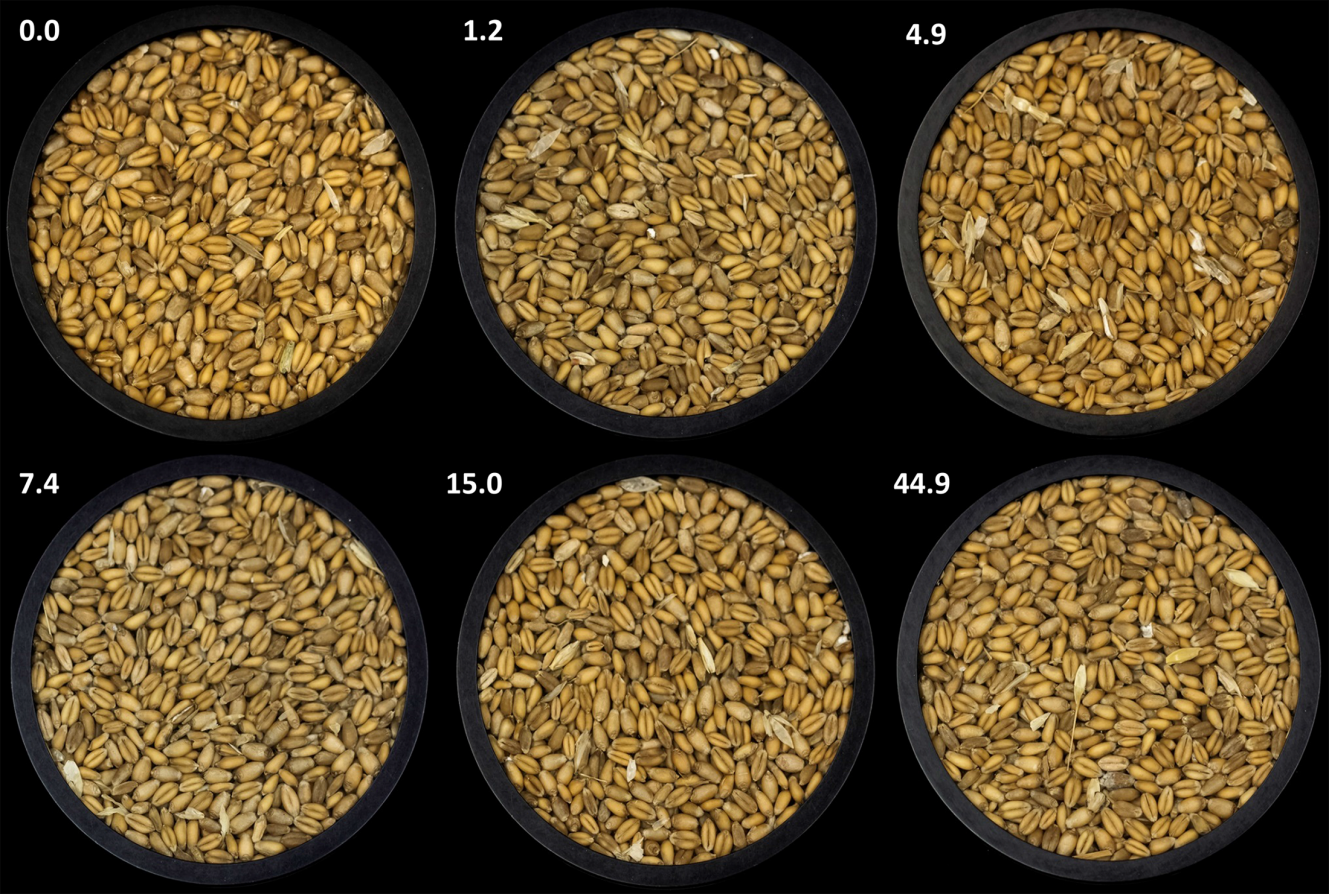
Winter wheat grain naturally contaminated with OTA at 0.0, 1.2, 4.9, 7.4, 15.0, 44.9 ng/g and collected during the 2011–2014 on-farm storage survey of Ontario winter wheat.

OTA-positive samples (LOD>0.3ng/g) were from 5 independent bins from 5 different farm locations comprising 2.2% of the total grain samples taken with only 3 samples (1.3% of the total grain samples taken) had OTA concentrations exceeding the EU regulation. Disregarding isolated grain clumps, no noticeable visual difference was observed between grain samples contaminated by OTA at various concentrations and visible growth by *P*. *verrucosum* (Fig 4).

### OTA incidence in on-farm storage bins

The five bins containing grain contaminated with OTA and one bin with a grain clump found positive for OTA were studied in greater detail (supplementary information S1 Table). All bins were located on different farms. No difference was found between on-farm bins (*df*_*n*_ = 5, *df*_*d*_ = 24) and OTA (*F* = 0.36, *p* = 0.8949), OTB (*F* = 0.70, *p* = 0.6495), and citrinin (*F* = 3.52, *p* = 0.578) levels (Table 5). A total of 10 grain clump, found near manhole opening and door frames; 11 debris and old grain samples, found in augers, on floor or attached to walls; and 16 grain samples from different areas in the bin were collected. OTA was found in all clump and debris samples (n = 21) and in only 31% (n = 5) of grain samples collected. OTA, citrinin and OTB co-occurred in 77% of positive samples (n = 20). OTB was not detected in 6 samples whereas OTA and citrinin co-occurred in all positive samples.

## Discussion

**Fig 5:**
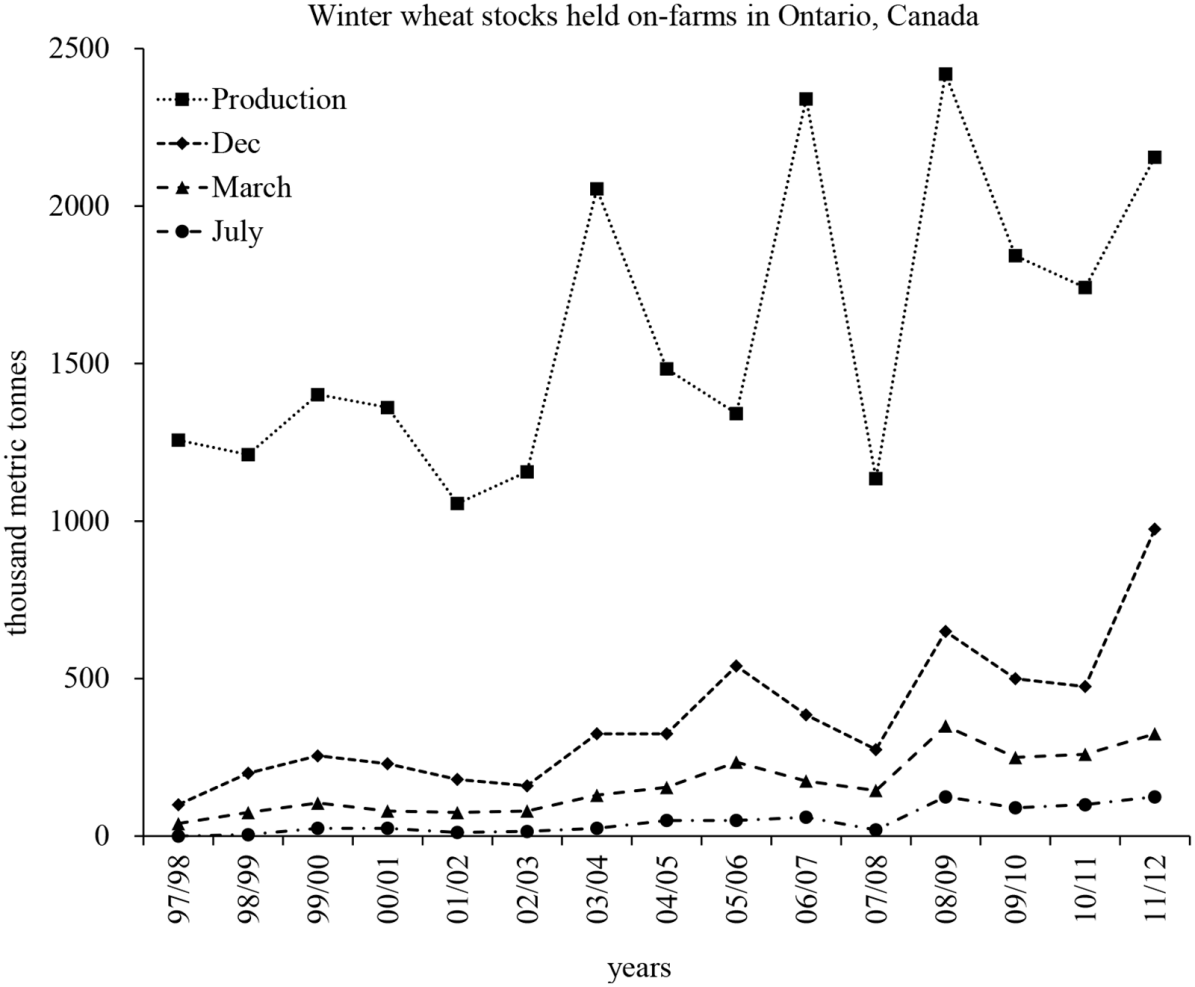
Winter wheat production and fluctuation of on-farm grain stocks held in Ontario, Canada from July 1997 to December 2012. Data acquired from Statistics Canada from 1997–2012. Source: Annual Field Crop Reporting Series, Stock of principal field crops.

Southwestern Ontario accounts for 73% of Canadian winter wheat production [1]. According to data collected from Statistic Canada [3], a 10-fold increase in the amount of wheat grain stored in December occurred in the last 15 years, growing from 0.1Mt in 1997 to approximately 1.0Mt in 2011 (Fig 5).

Approximately 44% and 11% of on-farm stored grain was kept until March and July of the following year, respectively. The current survey of on-farm storage was conducted from mid-December to the second week of April each year in 6 counties (Chatham-Kent, Middlesex, Lambton, Perth, Essex and Huron) where approximately 50% of the total winter wheat grain in the province was produced. [1].

Free standing cylindrical bins, made by bolting sections of corrugated galvanized steel, are the most common storage structure in Ontario. Typically, after drying the grain to straight grade (14.5% moisture content), farmers in Ontario use natural-air drying as a cost-effective method to reduce temperature gradient and moisture migration in storage bins to prevent the formation of hot spots and spoilage. Natural-air drying occurs when the relative humidity of the outside air is below the equilibrium moisture content of the grain. For instance, if wheat is aerated with outside air at 15°C or 0°C and 60% HR the equilibrium moisture content will be 12.4% and 13.5%, respectively [32]. Although, a flat-bottom bin equipped with a fully perforated floor above a plenum provides a uniform air distribution through bulk grain [33], debris, residue of old grain and fines accumulating in the space below the aerated floor can restrict or divert airflow risking the formation of non-aerated or stagnant zones with high moisture content [34].

The headspace over the grain bulk inside these bins experience large temperature and relative humidity fluctuations because of big surface area on the roofs of these bins and solar radiation. Even in small bins temperature gradients can induce slow convection currents causing grain moisture migration to the top center region in winter [35]. This movement of moisture can stimulate fungal growth resulting in aggregation of grains forming bridges across the top of the bin content [36]. Spoilage can often occur on the center top area of the bulk grain surface due to: a) insufficient aeration generating temperature gradients, moisture migration, condensation; and/or b) upward air movement through warm grain in cold weather causing under roof condensation that drips on top of the grain surface [33, 37].

A total of 18 hard clumps of spoiled grain with noticeable mold growth was collected during the 4 years of this survey, 10 of those were positive for OTA, citrinin and OTB with a mean±SE level of 207.1±108.3, 30.4±16.5 and 25.7±12.8 ng/g, respectively. The mean ratio of OTA:OTB and OTA:citrinin concentration were 8:1 and 7:1 ranging from 3:1 to 576:1 and 1:1 to 104:1, respectively. Although all clumps were collected from top and bottom man-access doors where free moisture from precipitation or condensation resulted in spoilage and heating, only 56% of samples were positive for toxins produced by *P*. *verrucosum* (Table 5). In the clumps, after the initial heating, different species interact, compete and dominate simultaneously and in succession until the clumps cool down and fungal growth declines [31]. The fungi prevailing at different stages in the development of the clump will depend on the inoculum, moisture (water activity) and when grain is moist, gas composition [38]. The condition in which *P*. *verrucosum* will dominate and the extent of OTA contamination, in the clump and in the area adjacent to it, requires further study. The distribution of OTA contamination in clumps was highly heterogeneous.

No sample collected from intermediate loads (aggregated grain samples collected from the auger during grain out-loading) or from disturbed surfaces (where grain had been partially removed during storage) tested positive for OTA. Grain samples (12 kg) positive for OTA were all from highly localized areas related to moisture translocation or migration resulting in condensation in the bin or areas where free moisture from precipitation including snow entered the bin. The main contributing factors to *P*. *verrucosum* growth and OTA accumulation in each sample are described, in decreasing level of concentration, as follows (Table 4): 1) A SWW sample (44.9 ng/g; 4514 CFU/g) collected from the top center under a snow cone of a non-aerated bin with an untouched frozen surface; 2) SRW sample (15.0 ng/g; 1014 CFU/g) collected from the top centre area of an undisturbed bin. In this bin, aeration was performed when air temperature was cold with low humidity resulting in water dripping from the in-loading grain downspout due to condensation from warm damp air rising through the grain mass. In winter, condensation dripping above the high moisture area of the bin (top center) might trigger spore growth that could lead to OTA accumulation. Improving air flow in storage bin roof vents [39] or blocking the gravity spouts during winter should prevent the escape of moist air through gravity downspouts; 3) A SRW sample (7.4 ng/g; 1313 CFU/g) collected from the top of the wagon representing the last grain flowing out of a non-aerated bin. Due to grain flow dynamics during unloading most of this grain came from the bottom of the bin, which would have included grain in the corners of the bin where the floor meets the wall; 4) A SRW/SWW blend sample (4.9 ng/g; 2450 CFU/g) collected from the first load coming out of a bin aerated every 6 weeks. Due to a funnel pattern that occurs during grain unloading much of this grain came from the top of the mass flowing through a center channel formed inside the grain mass [40, 41]; 5) A SWW sample (1.2 ng/g; 369 CFU/g) collected from south side of an untouched surface under a snow cap in a twice aerated bin. The snow came through the vents and the south exposure created opportunities for heating and melting that provided water activity conducive to the growth of *P*. *verrucosum* and OTA production.

Typically, during grain out-loading a funnel flow pattern occurs when discharging grain from storage bins resulting in the uppermost grain being discharged first flowing through a center channel formed inside the grain mass [40, 41] increasing the probabilities of finding spoilage pockets originated on the bulk grain surface in the first loads. In winter, water condensation above the high moisture area of the bin (top center) might trigger spore growth that could lead to OTA accumulation if *P*. *verrucosum* spores are present. Conversely, in fall and spring, the ambient temperature becomes higher than that of the center bulk and natural convection currents reverse direction causing buildup on the bottom of the bins thus increasing the probability of *P*. *verrucosum* activity and OTA contamination in the last loads.

*P*. *verrucosum* is able to grow and produce mycotoxins over a wide range of temperatures [42, 43], including low temperatures (0°C-4°C) [44, 45] especially when moisture is sufficiently high (20%) in a highly susceptible grain like rye [46]. OTA production is limited by a a_w_ level <0.80 (around 14% moisture content) [45]. More studies on incubation periods required for OTA formation under low temperatures in high moisture hot spots are needed in wheat. Hot spots of OTA contamination were most often in areas affected by moisture migration due to inadequate aeration and exposure to moisture from precipitation or condensation. These data indicate that different patterns of condensation and oxygen tension occur and this affects the nature and distribution of small pockets of toxin in a grain bin.

In our 4-year study of stored grain, we were not able to find multi-year OTA contamination in any of the on-farm storage bins surveyed. The lack of consistency of OTA contamination between years in the same storage bins following the same aeration patterns could indicate an interaction between harvest, post-harvest and storage management strategies with environmental factors that favors high grain moisture content at harvest and/or moisture migration and condensation inside grain bins. Fungal growth depends largely on temperature and water availability. Even within the closed conditions of a storage bin, large temperature and moisture gradients can occur causing heat-induced natural convection currents resulting in moisture migration within the stored grain bulk [35]. Although weather related factors were not considered in this study, due to the low thermal conductivity of grain [47] during winter the bulk of the grain retains heat while the periphery and air above the grain cools or warms to ambient temperature [33]. *P*. *verrucosum* growth and OTA accumulation in samples collected from untouched surfaces (n = 3) and the first load (n = 1) (Table 4) were most likely the result of moisture migration in winter. In the cold season, when the ambient temperature becomes lower than that of the center bulk, the warm bulk air rises into the headspace and condenses as it cools in contact with bin roof, leading to moisture accumulation that may both drip onto the top of the grain and pour down into the sides of the bins [37]. In spring or by the beginning of summer the ambient temperature becomes higher than that of the center bulk, and the natural convection currents reverse direction causing moisture buildup on the bottom of the bins [48], as is the case of samples collected from the last load.

Critical control points where OTA contamination can occur in storage bins were identified. In decreasing order of likelihood, they are: 1) grain clumps accumulated around or directly under manhole openings (n = 5, 370.3 ±198.1 ng/g; t = 7.2; p<0.0001); 2) debris and residue of old grain or grain clumps collected from the bin walls or left on storage floor (n = 6; 80.0 ±34.0 ng/g; t = 5.7; p<0.0001) and augers (n = 5; 59.7 ±30.7 ng/g; t = 5.0; p<0.0001); 3) grain clumps accumulated around side doors (n = 4; 46.3 ±19.0 ng/g; t = 4.9; p<0.0001). All clumps and debris samples collected from these bins were positive for OTA; 4) most grain samples collected and one clump sample (67%) collected from untouched surface (high moisture area on the central top part of grain bulk) were positive for OTA (n = 7; 15.9 ±8.0 ng/g; t = 2.9; p = 0.007); Conversely, 5) most samples (71%) collected from the first or last loads were negative for OTA (n = 27; 1.8 ±1.2 ng/g; t = 0.7; p = 0.514); and 6) all samples collected from disturbed surfaces and intermediate loads were negative for OTA (Table 5).

## Conclusion and recommendations

**Fig 6:**
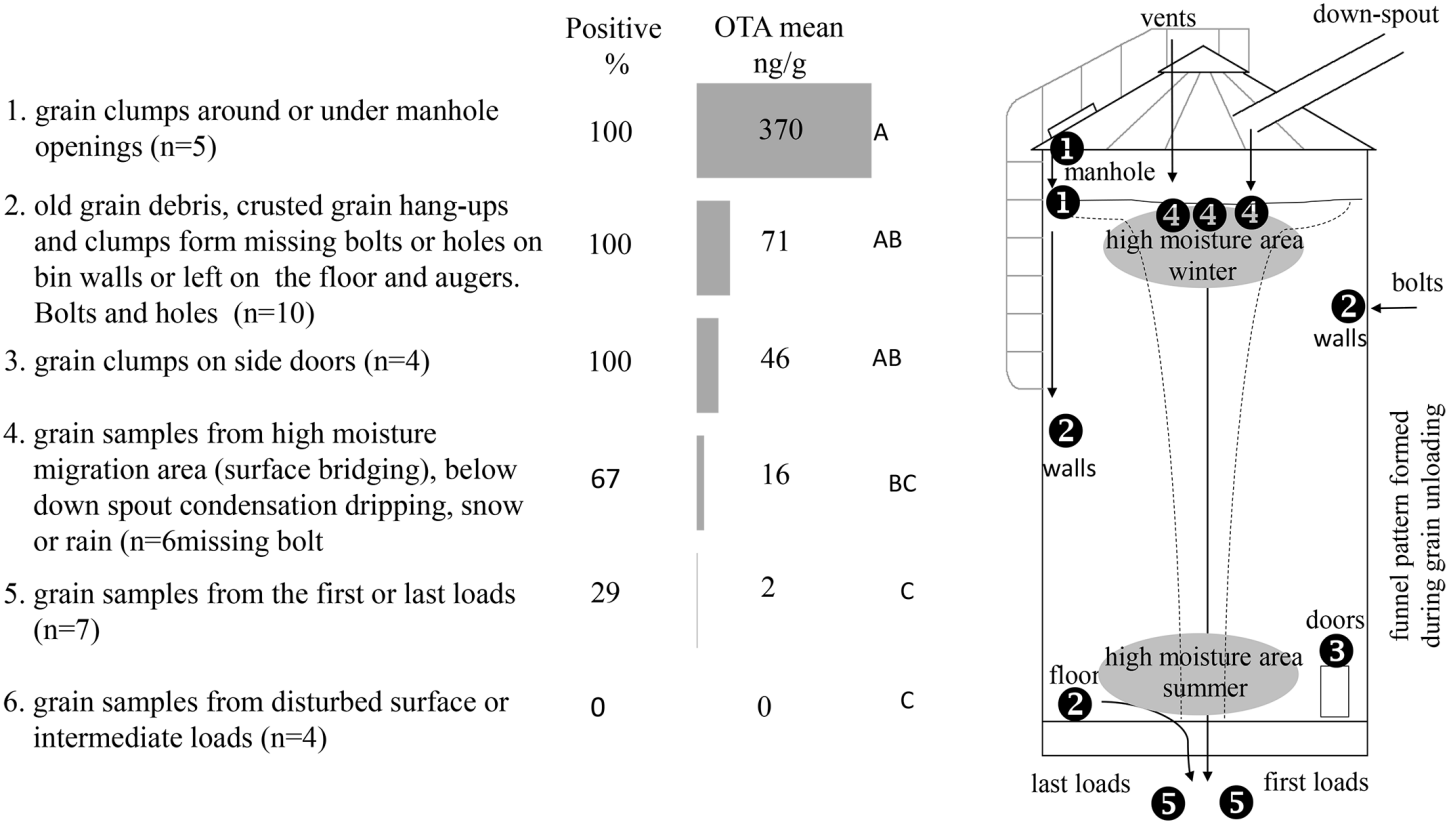
Critical point sources of OTA contamination in on-farm stored winter wheat.

OTA was rarely found in wheat samples in our survey. When found, it was highly-localized in areas of high moisture content in the grain bulk and clumps. We were surprised by the relationship between condensation and the occurrence of OTA in the colder periods of the storage cycle, and the isolated and specific nature of the pockets and clumps of contaminated grain (Fig 6).

This was true for new corrugated-steel bins and even when grain enters storage below the 14.5% threshold of moisture. Attention must be given with respect to bin design, construction and management. Designers and farmers must pay attention to the small details of managing the entry of liquid water moisture and the accumulation of condensation. The focus should be less on the movement of moisture out of the grain, and more on the removal of moisture as it leaves the grain in the head space to preventing condensation in or on the grain bulk. For example, in a modern new bin a missing bolt, a poorly sealed access door, an open down-spout are all potential sources of free moisture and condensation. The entry of snow in winter through vents and other openings is also of concern (Fig 6). We did not have the opportunity to sample from below the aerated floor, but suspect that the grain residue in these places are also contributors to inoculum and spoilage. Therefore, prevention is the key strategy through sanitation and bin inspection, to eliminate the sources of inoculum and free moisture.

## Supporting information

S1 Table *P*. *verrucosum* colony counts (CFU/g), mycotoxin occurrence (ng/g) and aeration practices associated with OTA positive grain, grain clumps and residue samples found during the 2011–2014 on-farm storage survey of Ontario winter wheat. CFU of *P*. *verrucosum* were determined by dilution plating on modified YES media (LOD: 63 CFU/g). OTA OTB and CTN were determined by LC-MS/MS with a LOD of 0.3, 0.5 and 0.6 ng/g, respectively.
(XLSX)
